# Supplementary material for: Surgical Outcomes of Video-Assisted versus Open Pneumonectomy for Lung Cancer: A Real-World Study
Source: Cancers (Basel). 2022 Nov 19;14(22):5683. doi: 10.3390/cancers14225683 (PMC9688192; doi:10.3390/cancers14225683)
Supplement: Supplementary file 1 [file cancers-14-05683-s001.zip › cancers-1986106-supplementary.pdf]

Supplementary Table S1. Baseline characteristics between open and VATS pneumonectomy group patients before propensity-score matching.

**Characteristic**		**Total**<br>N=583	**Open**<br>n=478(82.0)	**VATS**<br>n=105(18.0)	***p***
		No. (%)	No. (%)	No. (%)	
Sex	Male	517(88.7)	428(89.5)	89(84.8)	.174
	Female	66(11.3)	50(10.5)	16(15.2)	
Age, years		59.7±8.0	59.6±7.8	60.5±8.8	.273
% Predicted FEV_1		79.6±15.0	79.3±14.8	80.9±15.7	.321
% Predicted DLCO		85.5±19.2	86.1±19.5	82.9±18.0	.121
Neo-ACT		65(11.1)	58(12.1)	7(6.7)	.124
Comorbidity		76(13.0)	66(13.8)	10(9.5)	.238
Pathology	SCC	410(70.3)	340(71.1)	70(66.7)	.664
	ADE	95(16.3)	76(15.9)	19(18.1)	
	Others	78(13.4)	62(13.0)	16(15.2)	
cT stage	2	341(58.5)	276(57.7)	65(61.9)	.433
	3/4	242(41.5)	202(42.3)	40(38.1)	
cN stage	0	125(21.4)	101(21.1)	24(22.9)	.696
	1/2	458(78.6)	377(78.9)	81(77.1)	
pT stage	2	311(53.3)	248(51.9)	63(60.0)	.131
	3/4	272(46.7)	230(48.1)	42(40.0)	
pN stage	0	108(18.5)	86(18.0)	22(21.0)	.479
	1/2	475(81.5)	392(82.0)	83(79.0)	
pM stage	0	576(98.8)	471(98.5)	105(100)	1.000
	1	7(1.2)	7(1.5)	0	
pTNM stage	I	54(9.3)	38(7.9)	16(15.2)	.020
	II-IV	529(90.7)	440(92.1)	89(84.8)	
Laterality	Right	105(18.0)	77(16.1)	28(26.7)	.016
	Left	478(82.0)	401(83.9)	77(73.3)	

Abbreviations: FEV_1= forced expiratory volume in 1s, DLCO= diffusion capacity of lung for carbon monoxide, Neo-ACT=neoadjuvant chemotherapy, SCC=squamous cell carcinoma, ADE=adenocarcinoma, χ^2 test was calculated from logistic regression model stratified by trail. *P* value is for the comparison between Open and VATS group.

Supplementary Table S2. Baseline characteristics between open and VATS pneumonectomy patients after propensity-score matching.

**Characteristic**		Total N=203 No. (%)	Open n=132 No. (%)	VATS n=71 No. (%)	*p*
Sex	Male	177(87.2)	115(87.1)	62(87.3)	.967
	Female	26(12.8)	17(12.9)	9(12.7)	
Age, years		59.7±7.7	59.9±7.3	59.4±8.4	.638
% Predicted FEV_1		80.9±14.3	79.7±14.0	83.1±14.8	.107
% Predicted DLCO		86.6±17.9	87.7±18.4	84.5±17.1	.225
Neo-ACT		11(5.4)	7(5.3)	4(5.6)	1.000
Comorbidity		25(12.3)	16(12.1)	9(12.7)	.909
Pathology	SCC	138(68.0)	90(68.2)	48(67.6)	.308
	ADE	38(18.7)	27(20.4)	11(15.5)	
	Others	27(13.3)	15(11.4)	12(16.9)	
cT stage	2	127(62.6)	83(62.9)	44(62.0)	.899
	3/4	76(37.4)	49(37.1)	27(38.0)	
cN stage	0	41(20.2)	29(22.0)	12(16.9)	.391
	1/2	162(79.8)	103(78.0)	59(83.1)	
pT stage	2	125(61.6)	82(62.1)	43(60.6)	.828
	3/4	78(38.4)	50(37.9)	28(39.4)	
pN stage	0	29(14.3)	17(12.9)	12(16.9)	.435
	1/2	174(85.7)	115(87.1)	59(83.1)	
pM stage	0	130(98.5)	98(98.0)	32(100.0)	1.000
	1	2(1.5)	2(2.0)	0	
pTNM stage	I	18(8.8)	9(6.8)	9(12.7)	.161
	II-IV	185(91.2)	123(93.2)	62(87.3)	
Laterality	Right	45(22.2)	29(22.0)	16(22.5)	.926
	Left	158(77.8)	103(78.0)	55(77.5)	

Abbreviations: FEV_1= forced expiratory volume in 1s, DLCO= diffusion capacity of lung for carbon monoxide, Neo-ACT=neoadjuvant chemotherapy, SCC=squamous cell carcinoma, ADE=adenocarcinoma, χ^2 test was calculated from logistic regression model stratified by trail. *P* value is for the comparison between Open and VATS group.

Supplementary Table S3. Clinical characteristics and perioperative outcomes between right and left pneumonectomy via Open or VATS approach

**Characteristic**	**Open**			**VATS**		
	**Right n=77**	**Left n=401**	***p***	**Right n=24**	**Left n=61**	***p***
pT Stage			.218			.329
I/II	35(45.5)	213(53.1)		13(54.2)	40(65.5)	
III/IV	42(54.5)	188(46.9)		11(45.8)	21(34.4)	
Operative time, min	156.5±46.0	136.9±45.5	.001	209.6±59.7	188.6±73.7	.217
Blood loss, ml	261.0±350.2	212.6±236.0	.131	177.1±95.5	234.4±258.9	.226
Number of dissected LN	18.0±7.7	16.8±7.0	.147	17.9±5.5	18.2±9.2	.857
R0 Surgery, %	63(81.8)	376(93.8)	.000	22(91.7)	57(93.4)	.774
Major complications, %	19(24.7)	67(16.7)	.096	5(20.8)	5(8.2)	.104
Atrial Fibrillation	4(5.2)	38(9.5)		1(4.1)	2(3.3)	
Bleeding	5(6.5)	6(1.5)		0	1(1.6)	
ARDS	1(1.3)	9(2.2)		0	0	
Infection	2(2.6)	7(1.7)		0	2(3.3)	
Bronchopleural Fistula	5(6.5)	3(0.7)	.002	4(16.7)	0	.005
Chylothorax	1(1.3)	2(0.5)		0	0	
Esophageal injury	0	1(0.2)		0	0	
Gastrointestinal bleeding	1(1.3)	1(0.2)		0	0	
Days in ICU	2.9±2.6	3.0±4.3	.922	4.7±8.4	2.2±1.9	.172
LOS, day	12.4±8.4	11.6±9.4	.511	10.8±7.4	10.0±4.8	.672
30 days mortality, %	3(3.9)	6(1.5)	.336	1(4.2)	1(1.6)	1.000
90 days mortality, %	3(3.9)	8(2.0)	.546	2(8.3)	1(1.6)	.394

Abbreviations: LN=lymph node, LOS=length of stay. Categorical data are expressed as number (%) and continuous data as mean ± SD or median (interquartile range)
